# PET Index of Bone Glucose Metabolism (PIBGM) Classification of PET/CT Data for Fever of Unknown Origin Diagnosis

**DOI:** 10.1371/journal.pone.0130173

**Published:** 2015-06-15

**Authors:** Jian Yang, Xinxin Liu, Danni Ai, Jingfan Fan, Youjing Zheng, Fang Li, Li Huo, Yongtian Wang

**Affiliations:** 1 Beijing Engineering Research Center of Mixed Reality and Advanced Display, School of Optics and Electronics, Beijing Institute of Technology, Beijing, 100081, China; 2 Department of Nuclear Medicine, PET Center, Peking Union Medical College Hospital, Beijing, 100032, China; Shenzhen institutes of advanced technology, CHINA

## Abstract

**Objectives:**

Fever of unknown origin (FUO) remains a challenge in clinical practice. Fluorine-18 fluorodeoxyglucose (FDG) positron emission tomography/computed tomography (PET/CT) is helpful in diagnosing the etiology of FUO. This paper aims to develop a completely automatic classification method based on PET/CT data for the computer-assisted diagnosis of FUO.

**Methods:**

We retrospectively analyzed the FDG PET/CT scan of 175 FUO patients, 79 males and 96 females. The final diagnosis of all FUO patients was achieved through pathology or clinical evaluation, including 108 normal patients and 67 FUO patients. CT anatomic information was used to acquire bone functional information from PET images. The skeletal system of FUO patients was classified by analyzing the standardized uptake value (SUV) and the PET index of bone glucose metabolism (PIBGM). The SUV distributions in the bone marrow and the bone cortex were also studied in detail.

**Results:**

The SUV and PIBGM of the bone marrow only slightly differed between the FUO patients and normal people, whereas the SUV of whole bone structures and the PIBGM of the bone cortex significantly differed between the normal people and FUO patients. The method detected 43 patients from 67 FUO patients, with sensitivity, specificity, positive predictive value, negative predictive value, and accuracy of 64.18%, 95%, 93.48%, 72.73%, and 83.33%, respectively.

**Conclusion:**

The experimental results demonstrate that the study can achieve automatic classification of FUO patients by the proposed novel biomarker of PIBGM, which has the potential to be utilized in clinical practice.

## Introduction

Fever of unknown origin (FUO) is characterized by an increase in temperature to > 38.3°C on several occasions for more than 3 weeks; the etiology behind FUO cannot be diagnosed even after at least a week of hospital stay [1]. Despite recent advances in diagnosis, FUO remains a major medical problem with up to 53% undiagnosed cases [2]. Currently, more than 200 known reasons have been accounted for FUO [3]. On the basis of possible causes, FUO is mainly classified under four categories, namely, infection, neoplasm, non-infectious inflammatory disease, and unknown cause [4]. In clinical practice, timely and accurately identification the cause of FUO is very important for further effective treatment of the disease [5]. ^18^F-fluorodeoxyglucose (FDG) is a structural analog of 2-deoxyglucose and accumulates in cells with high glycolytic rates, such as neoplastic cells and activated inflammatory cells. Hence, ^18^F-FDG positron emission tomography (FDG-PET) can be utilized as a powerful non-invasive approach to detect various cancers according to the metabolic changes of human body [6]. ^18^F-FDG–PET (FDG-PET) has been used to detect the etiology of FUO in clinical practice. The simultaneous acquisition and alignment of the whole-body anatomical and functional images of PET/computed tomography (CT) allow physicians to accurately locate the lesions of the FUO patients [7].

Before 2011, almost all studies utilized 16% to 69% of FDG–PET or PET/CT scans for FUO diagnosis [8,9]. In 2011, Kubota et al. analyzed the diagnostic results for FUO, FDG-PET is helpful in 51% FUO patients, sensitivity 81%, and specificity 75% [10]. Seshadri et al. also compared FDG-PET scans with ^111^In-granulocyte scintigraphy in 23 patients with FUO. FDG-PET was helpful in 52% of patients with FUO. Sensitivity, specificity, PPV, and NPV of FDG-PET were 86%, 78%, 86%, and 78%, respectively [11]. In the study of Sheng et al., a final diagnosis was established for 36 (75%) patients with FUO, and the sensitivity was 89%, specificity was 33%, PPV was 80%, and NPV was 50% [12]. Pelosi et al. retrospectively included 24 patients with FUO who underwent FDG-PET/CT. Helpfulness in this study was 46% for FDG-PET/CT. In 2012, Pedersen et al. observed FDG-PET/CT to be helpful in 45% in a study of 22 patients with FUO [13]. Crouzet et al. investigated the role of FDG-PET/CT in 79 retrospectively included patients with FUO. Helpfulness of FDG-PET/CT was 57%, sensitivity was 98%, and specificity was 87% [14]. Kim et al. observed FDG-PET/CT to be helpful in 52%, sensitivity was 92%, and specificity was 23% in 48 patients with FUO. The percentage of FDG-PET/CT scans helpful in the diagnostic process of FUO patients ranges between 42% and 67% [15]. In 2014, Gijsbert investigated the diagnostic value of FDG-PET in children with FUO, for which the sensitivity and specificity of FDG-PET/CT for the patients were 80% and 78%, respectively [16]. Through studies statistics, overall helpfulness for all studies investigating FDG-PET/CT was 54%.

Despite the number of studies that investigated FDG-PET for FUO diagnosis, the heterogeneity of the diagnostic value of FDG-PET or PET/CT remains inconclusive for the effective diagnosis of FUO [14,15]. However, most studies using FDG-PET/CT suffered from selection bias and were often retrospective, which could also have influenced the better results of FDG-PET/CT. At the same time, almost all of studies are performed by a doctor or expert, and the process is very time-consuming and labor-intensive.

Bone scan index (BSI), which was first proposed by Massimo Imbriaco et al. in 1998, is an imaging biomarker that reflects the tumor burden on bone scintigraphy [17,18]. As the biomarker reflects the extent of metastatic activity, it is valuable for deciding the treatment procedures for patient. In addition, future developments in diagnostic imaging may provide specific methods (e.g., SPECT/CT and PET/CT) of analyzing skeletal lesions.

In clinical practice, FDG–PET images show that many patients diagnosed with FUO have hypermetabolic bone marrow; in addition, bone marrow glycolysis is activated at different levels in patients with infection, neoplasm, and non-infectious inflammatory disease due to tumor cell infiltration or some stimulation factors in blood. Most conventional diagnostic methods are time consuming and may lead to large measurement errors, especially for patients with diffusing or uneven increased metabolic activities. Therefore, this study proposes a novel method to quantify the metabolic activity of the bone marrow and introduces BSI as a PET Index of Bone Glucose Metabolism (PIBGM) to quantify the glucose metabolic activity of the bone. The purpose of this study is to study the glucose metabolic activity of the bone marrow differences between normal patients and FUO patients, and also, the principle of hypermetabolism for bones.

## Methodology

### 2.1 Patients

The protocol of this study (data collection and post-processing) was approved by the institutional review board of Peking Union Medical College Hospital. 175 patients were involved in the experiments. All these patients have given their written consent to the participation and received remuneration for it. We obtain informed written consent from guardians on behalf of the minors enrolled in our study. The ethics committees approve this consent procedure.

**Fig 1:**
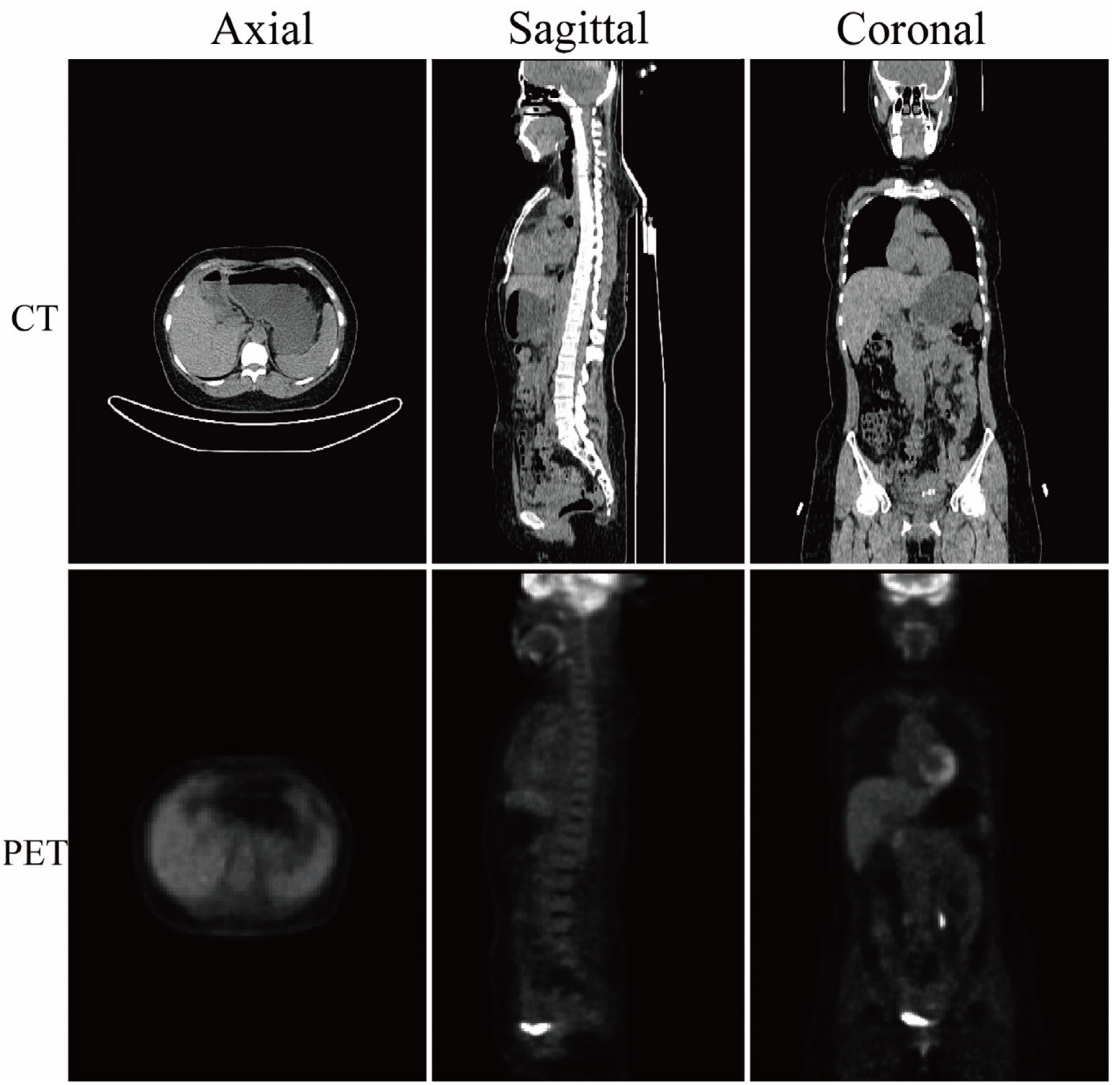
Example of the whole-body PET/CT images. The first and second rows correspond to the CT and PET images, respectively. The first to the third columns correspond to the axial, sagittal and coronal views of the 3D volume data, respectively.

From January 2008 to July 2013, the whole-body PET-CT datasets of 175 FUO patients at Peking Union Medical College Hospital were included for the experiments (Fig 1). For the datasets, there are totally 108 normal and 67 abnormal parents (Table 1), and the diagnosis conclusions are obtained according to strict physiological and biochemical examination. Moreover, the datasets include 79 males and 96 females with an age range of 13 to 75 years old (mean age: 47 years old). None of the FUO patients had an immunocompromised condition, a different spectrum of diseases causing fever, or nosocomial fever.

**Table 1:** Statistic of the patients.

DISEASE	INFLAMMATORY		INFECTION		NORMAL		NEOPLASM	
SEX	Female	Male	Female	Male	Female	Male	Female	Male
**COUNT**	8	12	16	13	44	64	11	7
**MINIMUM AGE**	18	20	17	14	24	25	28	13
**MAXIMUM AGE**	58	75	69	67	75	73	67	64

### 2.2 ^18^F-FDG PET/CT imaging

After at least 6 hours of fasting, the patients received an intravenous injection of FDG at 5.5 MBq/kg to 7.4 MBq/kg of body weight. All patients were checked for blood glucose levels before FDG injection. No patient had blood glucose levels greater than 160 mg/dL. Whole-body PET/CT scans were acquired using a combined PET/CT scanner (Siemens Biograph Sensation 64, Germany). And the images covered the body ranges from the meatus of the ear to the mid-thigh. Approximately 1 hour after FDG injection, PET/CT was conducted. Data acquisition was initiated by CT scanning from the head to the mid-thigh with 120 kV, 140 mAs. Tube rotation times of 0.5 second and 5 minutes were used, and the section thickness was matched to the PET section thickness. Immediately after CT scan, a whole-body emission PET scan was obtained with a 2 minutes acquisition per bed position using a 3D acquisition mode.

### 2.3 Clinical evaluation of PET/CT findings

This study synthetically summarizes all the examination results of the patient during hospitalization or outpatient care [12], which is hence further utilized to perform the retrospectively evaluation of the abnormal PET/CT images by the treating physician. Moreover, all the diagnosis results are determined according to the positive serologic assays, biopsy, surgery, or autopsy (Table 1). The etiology of FUO can be divided into three classes, namely, infection, malignance, and non-infectious inflammatory disease. Only patients with final diagnoses were selected for analysis.

### 2.4 Image Analysis

We presented a simple, robust, and completely automated whole-body bone segmentation algorithm for bone region extraction. This algorithm combines the automatic threshold segmentation algorithm and the enhanced Chan-Vese AC (CV-AC) algorithm for bone segmentation. The proposed image analysis is composed of four main procedures, namely, image interpolation, bed removal, automatic thresholding, and bone segmentation.

#### A. Image Interpolation

The CT and PET image resolutions for the obtained data sets are 512 × 512 and 168 × 168, respectively. The resolution difference in these two data sets may greatly interfere with the accuracy of bone extraction. If the resolution of the CT image is downgraded to 168×168, the information of the bone is considerably degenerated. Hence, the PET image needs to be interpolated to the resolution of 512 × 512. In this study, we utilized linear interpolation to increase the resolution of the PET images to 512 × 512. Each pixel of PET image was extended into 3 × 3 pixels with the same gray value, forming a 504 × 504 resolution of PET image. Then, the four sides of the PET image were extended with zero value pixels, and the resolution of the PET image was extended to 512 × 512.

#### B. Bed Removement

The bed can absorb X-ray radiation and is inevitably included in the CT image; this inclusion of the bed induces large interference in the image quantification. Thus, the automatic bed removal in the CT image is imperative to accurately quantify the disease. The PET image can be utilized as a mask to remove the bed intensity in the CT image because the PET and CT images were already co-registered and the bed cannot be embedded in the PET image. The image was smoothed by Gaussian filter with a comparative large kernel, and a simple thresholding method was applied to the image to obtain background and human tissues. The outline of human tissues was easily extracted because of the homogenous background of the PET image. After obtaining the outline of the human tissue, a binarized PET image was utilized as a mask to multiply with the CT image. Then, the image intensities outside the human tissue were effectively removed.

#### C. Automatic Thresholding

Automatic thresholding aims to pre-segment the bone structure from other tissues based on the statistical analysis of the histogram of the whole image intensity distribution [19]. In this study, the histogram of the whole-body CT image was considered as the combination of three Gaussian functions that represent the intensities of fat, muscle, and bone. The Gaussian peak of bone was submerged in the distributions of fat and muscle because bone structure only accounted for a small part of the whole body. This study assumed that the obtained intensity distribution of the whole body is combined by the two Gaussian peaks of fat and muscle. Thus, segmentation can be achieved by the Gaussian function estimation of fat and muscle. Generally, the combined Gaussian function *y*(*x*) can be defined as follows:

$$y\left(x\right)=A_{m}e^{-{\left(\frac{x-B_{m}}{c_{m}}\right)}^{2}}+A_{f}e^{-{\left(\frac{x-B_{f}}{c_{f}}\right)}^{2}}$$ (1)

where *m* and *f* are the indices of muscle and fat, respectively. The best threshold can be determined as follows:

$$Threshold=B_{m}+\frac{\omega }{2}\times \left(B_{m}-B_{s}\right)$$ (2)

where *ω* is the weighting coefficient.

#### D. Bone segmentation

The active contour (AC) model is a framework for extracting the outline of an object from a noisy background. The AC model minimizes the energy function constructed by the combination of predefined internal and external energies. Internal energies are usually designed to preserve the smoothness of contour deformation, whereas external energies are designed to drive contour deformation toward the target boundary of the desired object [20]. The AC model is advantageous because it constantly guarantees the close and continuous form of the contour and does not require post-processing tasks to connect discontinued edges.

We developed the CV-AC algorithm to accurately segment the bone structures. *C* represents the evolving curve in the image plane Ω ⊂ *R* ^2^, *inside*(*C*) denotes the bone region, and *outside*(*C*) denotes the non-bone region. *I* represents the image intensity. Then, the segmentation procedure assumes to find two areas with approximately constant intensities. Hence, the energy function of the deformable model can be defined as [21]:

$$F_{1}\left(C\right)+F_{2}\left(C\right)={\int_{inside(C)}\left|I\left(x\right)-c_{1}\right|}^{2}dx+{\int_{outside\left(C\right)}\left|I\left(x\right)-c_{2}\right|}^{2}dx$$ (3)

where c_1_ and c_2_ are average intensities inside and outside the bone region, respectively.

The traditional CV-AC algorithm is robust to noise but sensitive to image contrast. The CV-AC algorithm may fall into a local minimum when bone intensities are largely inhomogeneous. Thus, we reformulated the energy function as follows:

$$F_{1}\left(C\right)+F_{2}\left(C\right)={\int_{inside(C)}\left|I\left(x\right)-c_{1}\right|}^{2}+\varepsilon \times {\left|I\left(x\right)-Thresh\right|}^{2}dx+{\int_{outside\left(C\right)}\left|I\left(x\right)-c_{2}\right|}^{2}+\varepsilon \times {\left|I\left(x\right)-Thresh\right|}^{2}dx$$ (4)

where *ε* and *Th* are the weighting coefficient and the predefined local threshold, respectively. By introducing the local adaptive threshold, the algorithm can automatically and adaptively segment the boundaries of bones.

The bone tissue extracted from the CT image can be utilized to extract corresponding intensity distributions in the PET image. We set interpolated PET image as the original image and CT image bone segmentation result as the mask. Through masking operation, we can extract bone metabolic information in the PET image. Such method can guarantee the full resolution utilization of the PET image and the full resolution of bone segmentation in the CT image.

#### E. Segmentation Evaluation

**Fig 2:**
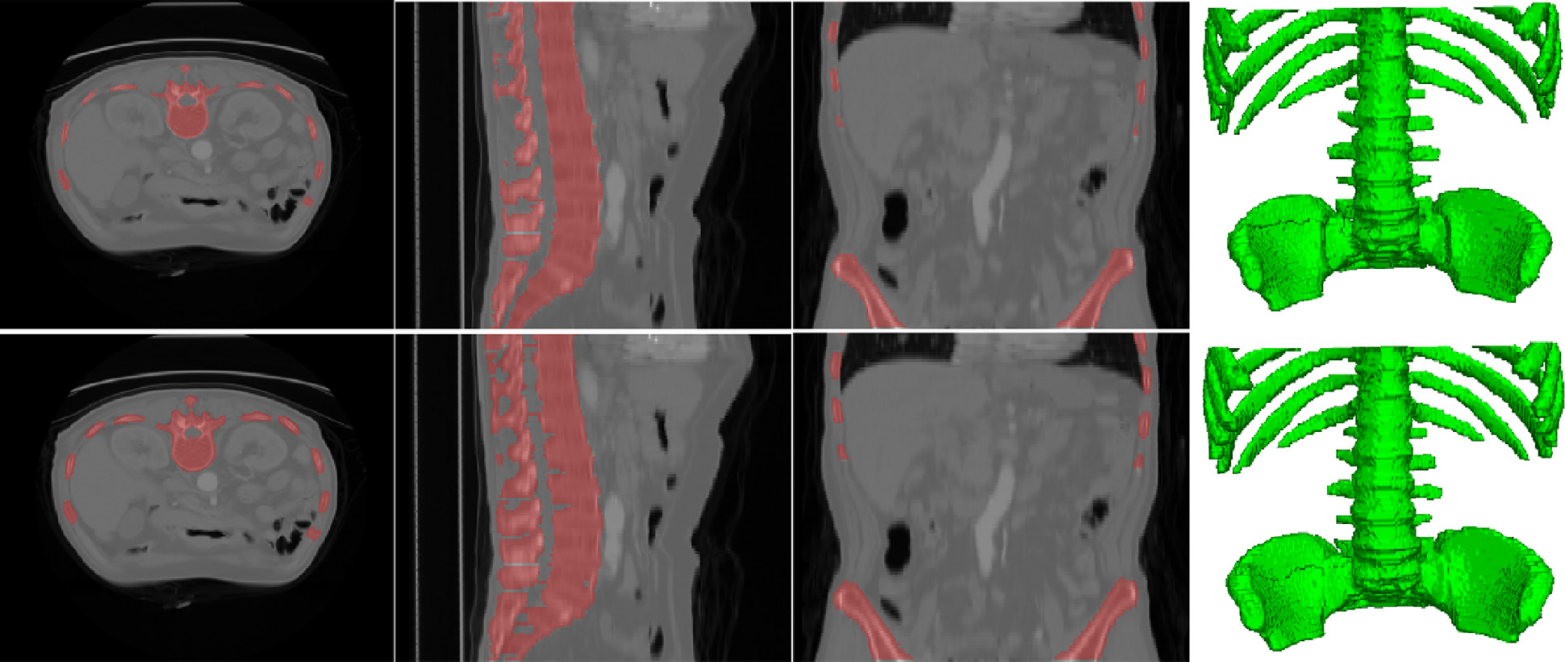
Example of the segmentation result and the corresponding ground truth. The first and the second row are the ground truth and the segmentation result by the propose method for a randomly selected patient. The first to the fourth columns are the axial, sagittal, coronal, and 3D render views of the bones superposed on the source CT data.

**Fig 3:**
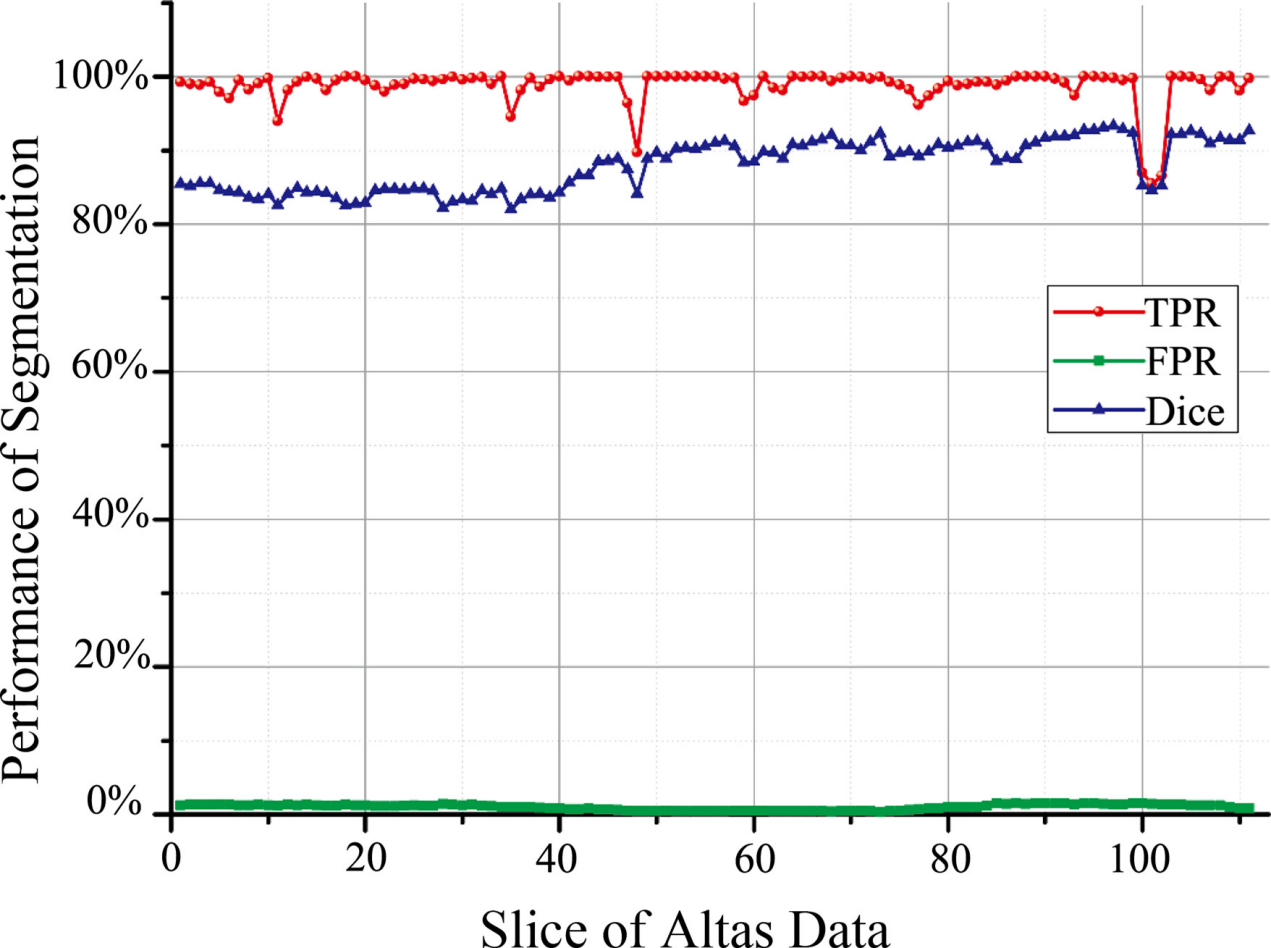
The performance of the proposed segmentation algorithm for all the data sets. The red, blue and green lines correspond to the true positive rate (TPR), the false positive rate (FPR), and the Dice coefficient (Dice).

To evaluate the performance of the proposed segmentation method, we performed bone segmentation on the data set provided by Surgical Planning Laboratory, Harvard Medical School in 2012. For this data set, all the bone structures were manually segmented by professionals and set as the golden standard for evaluating the performance of segmentation algorithms. The dimension of the data was 256 × 256 × 113, which ranged from the chest to the hips. Then, the proposed segmentation method was evaluated on the data set. Fig 2 compares the segmentation results of the ground truth (first row) and the proposed method (second row). It is obvious that the proposed segmentation method was effective for the segmentation of bone structures from the evaluation data set. Fig 3 provides the quantification results obtained through commonly used evaluation criteria, including true positive rate (TPR), false positive rate (FPR), and Dice coefficient. The mean TPR, FPR, and Dice coefficient of 113 slices of segmentation results were 0.988 ± 0.025, 0.0097 ± 0.0037, and 0.88 ± 0.034, respectively. These results demonstrate that our proposed segmentation method provides satisfactory results for the segmentation of the bone structures from CT volume data.

The first and the second rows correspond to the ground truth and the proposed method. The first to the fourth columns correspond to the axial view, sagittal view, coronal view and the volume rendering results.

### 2.5 SUV Calculation

Considering that the bone structure was extracted from the PET image, we can calculate the SUV of the bone structures of the whole body [22,23]. In general, the SUV is calculated on the basis of body weight and denoted as SUV_bw_. However, SUVs have also been calculated on the basis of body surface area (BSA) and lean body mass (LBM), and denoted as SUV_bsa_ and SUV_lbm_, respectively. The three SUVs can be calculated as follows:

$$SUV_{bw}=\frac{tissueconcentration\left(MBq/mL\right)}{injecteddose(MBq)/bodyweight(g)}$$ (5)

When the BSA of the patient was used for correction, SUV_bsa_ was formulated as follows:

$$SUV_{bsa}=\frac{tissueconcentration(MBq/ml)}{injecteddose(MBq)/BSA(m^{2})}$$ (6)

where BSA was calculated as follows:

$$BSA(m^{2})=0.007184\times bodyweight{\left(kg\right)}^{0.425}\times bodyheight{\left(cm\right)}^{0.725}$$ (7)

When the LBM was introduced for correction, SUV_lbm_ was formulated as follows:

$$SUV_{lbm}=\frac{tissueconcentration(MBq/ml)}{injecteddose(MBq)/LBM(kg)}$$ (8)

where the formulas of LBM for males and females differed and were calculated as follows:

$$LBM\left(female\right)=\left(1.07\times bodyweight\right)\left(kg\right)-148\times {\left[bodyweight\left(kg\right)/bodyheight\left(cm\right)\right]}^{2}$$ (9)

$$LBM\left(male\right)=\left(1.1\times bodyweight\right)\left(kg\right)-120\times {\left[bodyweight\left(kg\right)/bodyheight\left(cm\right)\right]}^{2}$$ (10)

### 2.6 PIBGM Calculation

PIBGM was combined with SUV to quantify PET image changes with regard to bones to estimate the inducibility of malignancy as well as the regional distribution of foci in the bones [24]. SUV was used to evaluate malignancy in the bone tissue, and then the affected proportion of bone tissue was calculated according to PIBGM. Malignancy was considered present when PET voxels had SUVs greater than a predefined threshold (t). Then, we counted the number of abnormal PET image voxels and calculated the proportion of abnormal voxels in all the bone structures. Hence, the PIBGM value can be calculated by the following equation:

$$PIBGM_{}=\frac{N_{t}\left|P_{i}>t\cap P_{i}\in bone\;tissue\right|}{N_{b}\left|P_{i}\in bone\;tissue\right|}$$ (11)

where *t* is predefined SUV threshold, *i* is the voxel index of PET data, *P*_*i*_ is the SUV of the *ith* voxel, *N*_*b*_ is the total number of bone tissue voxels, *N*_*t*_ is the total number of bone pixels with SUV larger than the predefined threshold.

### 2.7 Diagnostic Accuracy Quantification

In this study, we utilized sensitivity, specificity, positive predictive value (PPV), negative predictive value (NPV), and accuracy as the basic measures to quantify the diagnostic accuracy of the analyzed data sets. Sensitivity denotes the probability of a positive test in the presence of the disease of interest; this index is generally utilized to quantify the capability of a method to correctly identify subjects with the disease of interest [25,26]. According to statistical hypothesis theory, sensitivity, specificity, PPV, and NPV can be calculated by determining the true positive, true negative, false positive, and false negative.

## Results

### 3.1 SUV Analysis

This study analyzed 175 patients. Bone extraction and data analysis consumed 25.565 h in total, with an average of 8 min and 45 s for each patient. The proposed method involved the extraction of bone structures from the CT image; these structures were classified as marrow or cortex according to their intensity distribution. The segmented bone structures were utilized to extract the corresponding structures in the PET image. The SUV_bw_, SUV_bsa_, SUV_lbm_, and PIBGM for all the bone structures were statistically quantified (Table 2). Table 2 showed that the SUV of the bone was slightly higher in the FUO patients than in the normal people. The SUVs were higher in the neoplasm and non-infectious inflammatory disease patients than in the infection patients and normal people (Table 2). Therefore, the three etiologies of FUO may enhance bone glucose metabolism. However, SUV_bw_, SUV_bsa_, and SUV_lbm_ presented no significant difference for the discrimination abilities.

**Table 2:** Comparative analysis of mean and p-value with respect to SUV_bw_, SUV_bsa_ and SUV_lbm_ for all the patients.

Patients	Non-Infectious Inflammatory		Infection		Neoplasm		Normal
	Mean	p-value	Mean	p-value	Mean	p-value	Mean
**SUV_bw_**	1.28±0.26	0.1163	1.23±0.38	0.02772	1.21±0.39	0.2636	1.10±0.43
**SUV_bsa_**	0.37±0.06	0.05932	0.35±0.12	0.003093	0.34±0.12	0.09206	0.28±0.12
**SUV_lbm_**	1.03±0.18	0.08168	0.99±0.30	0.0102	0.97±0.31	0.1665	0.83±0.33

**Fig 4:**
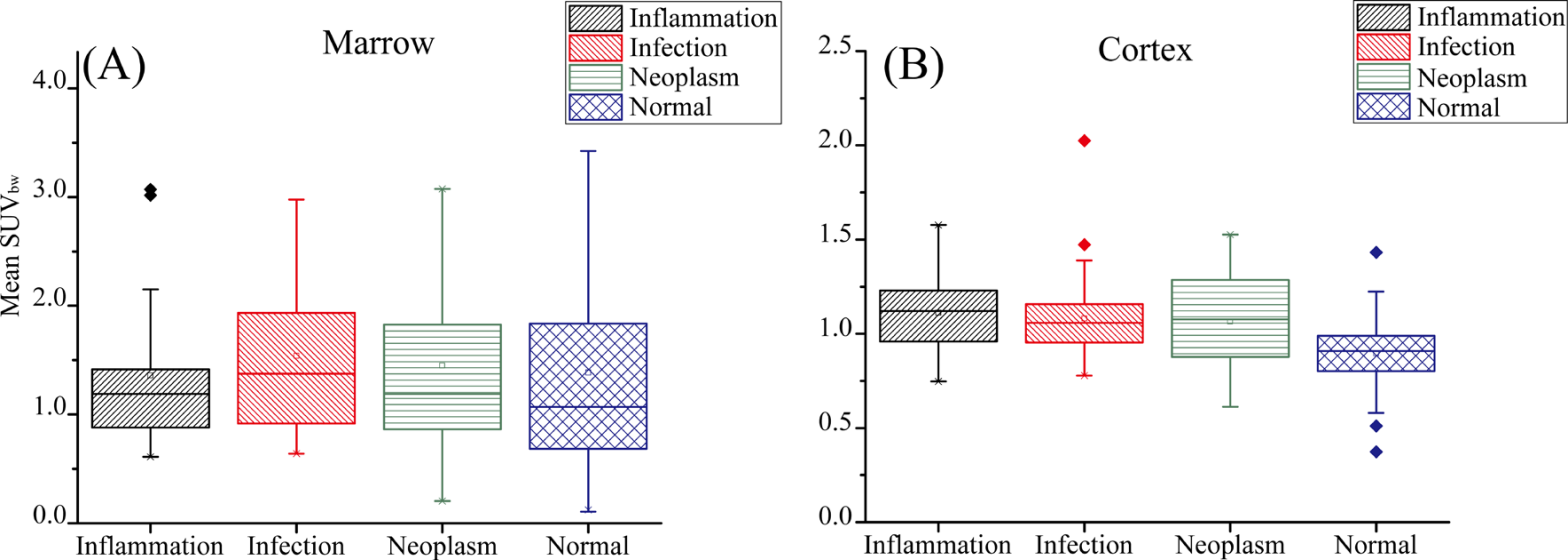
Boxplot of the mean value of SUV_bw_ for all the data sets of the Inflammation, Infections, Neoplasm, and Normal patients. (A) and (B) correspond to the statistic distributions of bone marrow bone cortex, respectively.

The mean SUV_bw_ values for non-infectious inflammatory, infection, malignancy, and normal people were 1.34, 1.16, 1.56, and 0.93 for the marrow, respectively, and 1.06, 1.03, 1.09, and 0.86 for the cortex, as shown in Fig 4. The mean SUV_bw_ of the marrow was significantly higher than that of the cortex. Although the SUV_bw_ values for the marrow were more dispersed than those for the cortex, no apparent differences were still observed between the normal people and FUO patients. Moreover, the SUV_bw_ values were more dispersed for the neoplasm and non-infectious inflammatory disease patients than for the infection patients and normal people.

The discrimination abilities of the normal people and FUO patients were compared through the t-test of SUV_bw_ values (Table 3). The p values for the cortex of the non-infectious inflammatory disease, infection, and neoplasm patients were 0.0002, 0.0013, and 0.0645, respectively. Compared with the patients with infection (p = 0.0645), the patients with non-infectious inflammatory disease and neoplasm presented larger differentiability (p = 0.0002, 0.0013). The SUV_bw_ values for the bone marrow did not significantly differ between the normal people and FUO patients with non-infectious inflammatory disease, infection, and neoplasm (p = 0.8884, 0.4002, 0.7606). The SUV analysis at the bone marrow revealed no significant difference between the normal people and FUO patients. Although the SUV_bw_ for the cortex was effective in differentiating non-infectious inflammatory disease and infection patients from normal people, it cannot be utilized to differentiate neoplasm patients.

**Table 3:** Comparative analysis of the discrimination abilities of cortex and marrow of the proposed method for Normal and FUO patients by using t-test.

Patients	Bone Cortex		Bone Marrow	
	t	p-value	t	p-value
**Inflammatory**	4.15	0.0002	-0.14	0.8884
**Infection**	3.40	0.0013	0.85	0.4002
**Neoplasm**	1.94	0.0645	0.31	0.7606

To further investigate the metabolic activities of the bone cortex in FUO patients, we introduced the parameter discriminative ability for the normal person (DANP). This parameter specifies the percentage of successfully identified normal persons. The FDG-PET result of a patient was considered abnormal when the mean SUV_bw_ was greater than the thresholds (according to statistical result, the experts decided) and normal when the mean SUV_bw_ was less than the thresholds. The recognition ability of the proposed method for non-infectious inflammatory, infection, and neoplasm rapidly decreased with decreasing DANP, the results are shown in Table 4. When the DANP was set to an acceptable ratio of 95%, the identification capabilities of the method for inflammatory, infection, and malignancy were 45%, 27.59%, and 44.44%, respectively. These data indicate that the widely used SUV standard is not good enough to identify FUO patients.

**Table 4:** Comparative analysis of the recognition accuracies of FUO patients with respect to different DANP values.

DANP	Threshold of *SUV*_*bw*_	Inflammatory	Infection	Neoplasm	Sensitivity	Specificity	PPV	NPV
100%	1.43	5%	6.90%	11.11%	100%	63.53%	7.46%	100%
95%	1.14	45%	27.59%	44.44%	83.33%	71.03%	37.31%	95%
90%	1.09	55%	41.38%	44.44%	73.81%	72.93%	46.27%	90%
85%	1.02	65%	51.72%	61.11%	70.91%	77.31%	58.21%	85%

### 3.2 PIBGM Analysis

**Fig 5:**
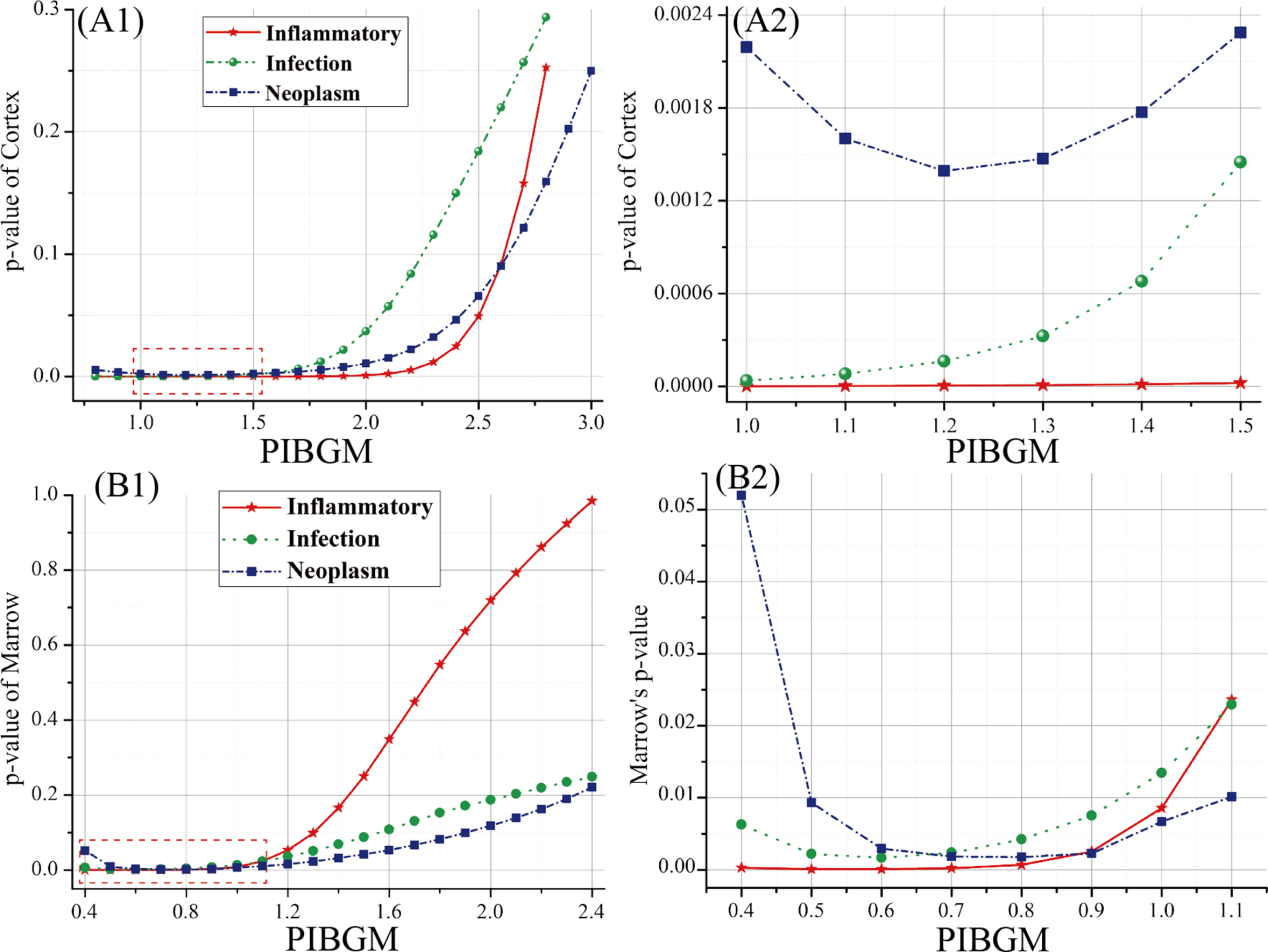
Distribution between p-value and the PIBGM for all the patients. (A1) and (A2) show the p-value of cortex and the corresponding magnified view with PIBGM values of 1.0 to 1.5. (B1) and (B2) show the p-value of marrow and the corresponding magnified view with PIBGM values of 0.4 to 1.1.

As aforementioned, the SUVs did not significantly differ among the three classifications of FUO. Consequently, we introduced PIBGM to further analyze the metabolic activities of bones and classify the disease. The thresholds of PIBGM were set in the range of 0.4 to 2.2 with an increasing step of 0.2. According to the t-test values, we can confirm that the differentiation capability of the proposed method was better for the cortex than for the marrow in all the three classes of patients (Table 5 and Fig 5). The p values for all FUO patients were comparatively constant with values of 0 to 0.024 in the PIBGM threshold range of 0.9 to 1.5. However, the p values rapidly increased as the PIBGM threshold increased from 1.6 to 3.0. This result indicates that the PIBGM threshold range of 0.9 to 1.5 is a good criterion to differentiate normal people from FUO patients. In such ranges, the PIBGM values for the cortex significantly differed between the normal patients and FUO patients.

**Table 5:** Comparative analysis of p-values with respect to different values of PIBGM for FUO patients by using t-test.

PIBGM	Inflammatory		Infection		Neoplasm	
	Cortex	Marrow	Cortex	Marrow	Cortex	Marrow
**0.4**	0.0000	0.0003	0.0001	0.0063	0.5212	0.0520
**0.6**	0.0000	0.0001	0.0000	0.0017	0.0422	0.0030
**0.8**	0.0000	0.0007	0.0000	0.0042	0.0054	0.0018
**1.0**	0.0000	0.0086	0.0000	0.0135	0.0022	0.0067
**1.2**	0.0000	0.0531	0.0002	0.0359	0.0014	0.0155
**1.4**	0.0000	0.1660	0.0007	0.0683	0.0018	0.0318
**1.6**	0.0000	0.3488	0.0031	0.1082	0.0030	0.0534
**1.8**	0.0002	0.5474	0.0120	0.1527	0.0055	0.0821
**2.0**	0.0010	0.7194	0.0369	0.1872	0.0107	0.1185
**2.2**	0.0052	0.8608	0.0839	0.2190	0.0222	0.1628
**2.4**	0.0248	0.9845	0.1498	0.2485	0.0464	0.2207

**Fig 6:**
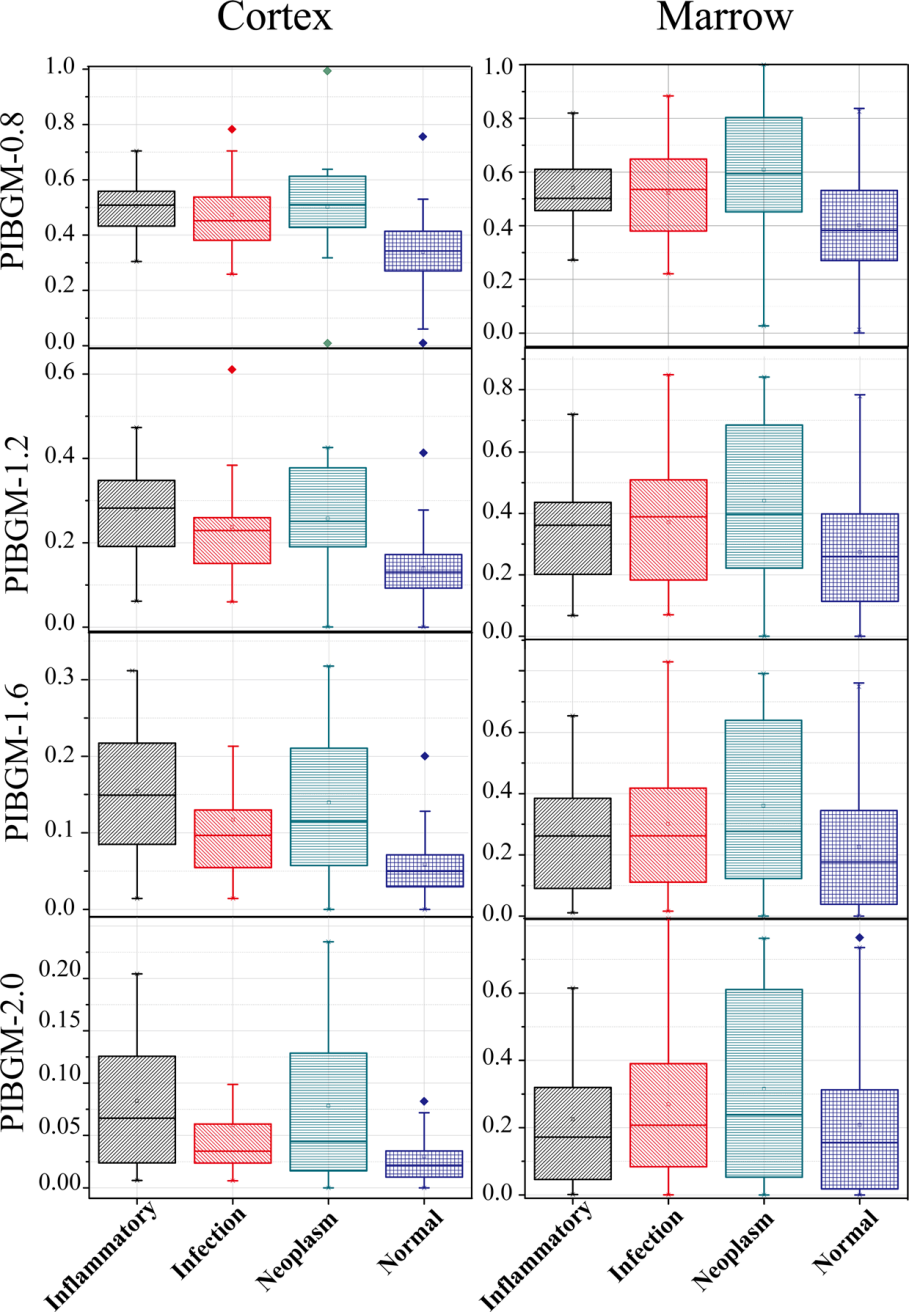
Boxplot of PIBGM with different SUV_bw_ thresholds. The first to the fourth rows show the boxplot of PIBGM with the thresholds of 0.8, 1.2, 1.6, and 2.0. While the first to the second rows correspond to the distributions of cortex and marrow.

The PIBGM values for the marrow scattered in large ranges for all the four classes of patients. With increasing PIBGM threshold from 0.8 to 2.0, the scattering ranges slowly contracted. Hence, no significant differences in the PIBGM values at the bone marrow existed between the normal people and FUO patients (Fig 6). By contrast, the cortex PIBGM dispersion range of the normal people was considerably lower than that of the FUO patients. When the thresholds were larger than 1.6, the dispersion ranges of the infection patients were roughly submerged in that of the normal patients. This result indicates that the discriminant condition with thresholds between 0.8 and 1.2 of the cortex is more reliable than the other values.

**Fig 7:**
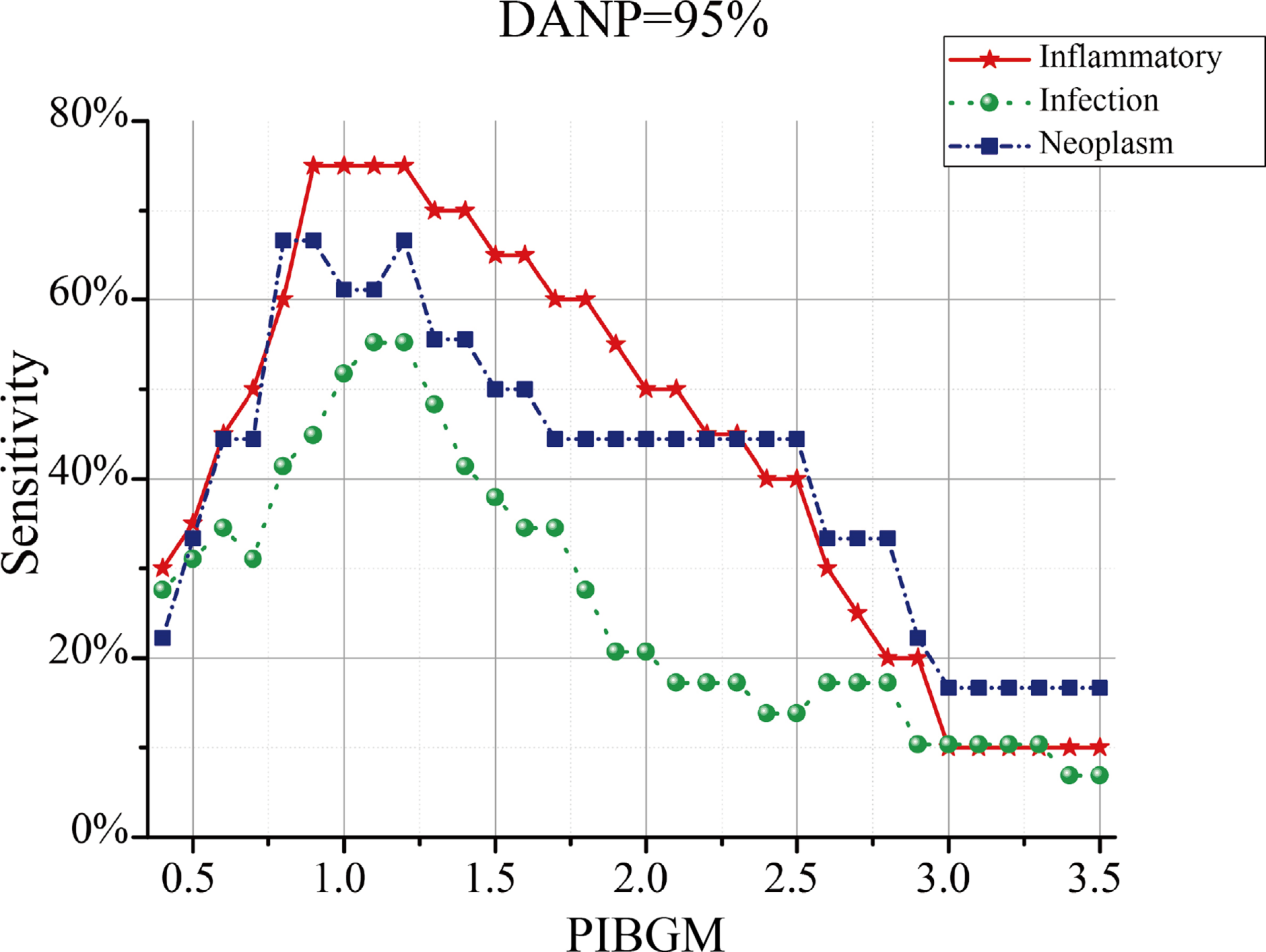
Distribution between Sensitivity and PIBGM for patients with discriminative ability of 95%. The red, green and blue lines correspond to inflammation, infection and neoplasm patients.

The sensitivities of the proposed method for non-infectious inflammatory disease, infection, or neoplasm presented a convex shape (Fig 7). When the PIBGM threshold was set to 1.2, the highest sensitivities of the proposed method for non-infectious inflammatory disease, infection, and neoplasm were 75%, 55.17%, and 66.67%, respectively.

**Fig 8:**
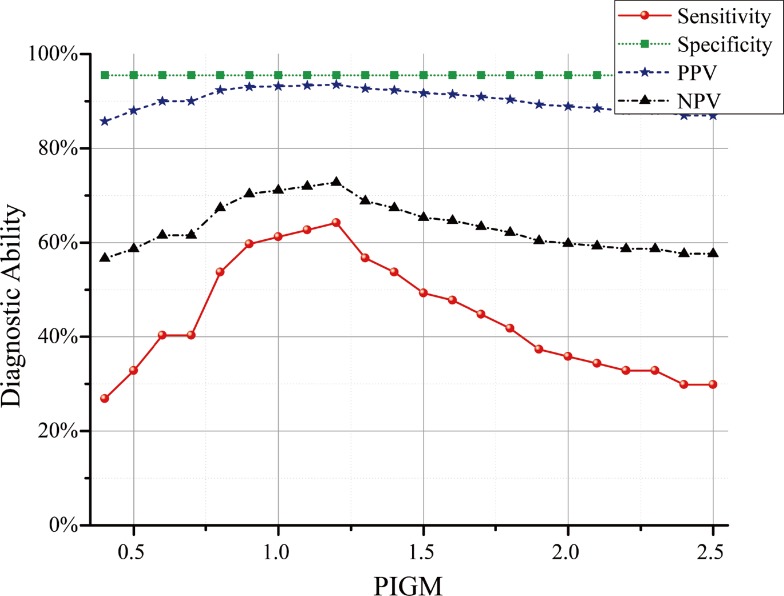
Relationship between the diagnostic ability and PIBGM for cortex. The red, green, blue, and black lines correspond to the sensitivity, specificity, positive predictive value (PPV), and negative predictive value (NPV), respectively.

Specificity had with a constant value of 95% for the various PIBGM threshold values. Sensitivity presented a convex shape; that is, it initially increased and then peaked to 64.18% when the PIBGM threshold was 1.2 and then gradually decreased to 30%. When the PIBGM threshold was 2.5. By contrast, the PPV ranged from 70% to 93.48%, with the peak reached at the PIBGM threshold of 1.2. The NPV gradually increased from 56.64% to 72.73% at the PIBGM threshold of 1.2 and then decreased. These data suggest that 1.2 is the best PIBGM threshold for the three groups of FUO patients (Fig 8).

**Fig 9:**
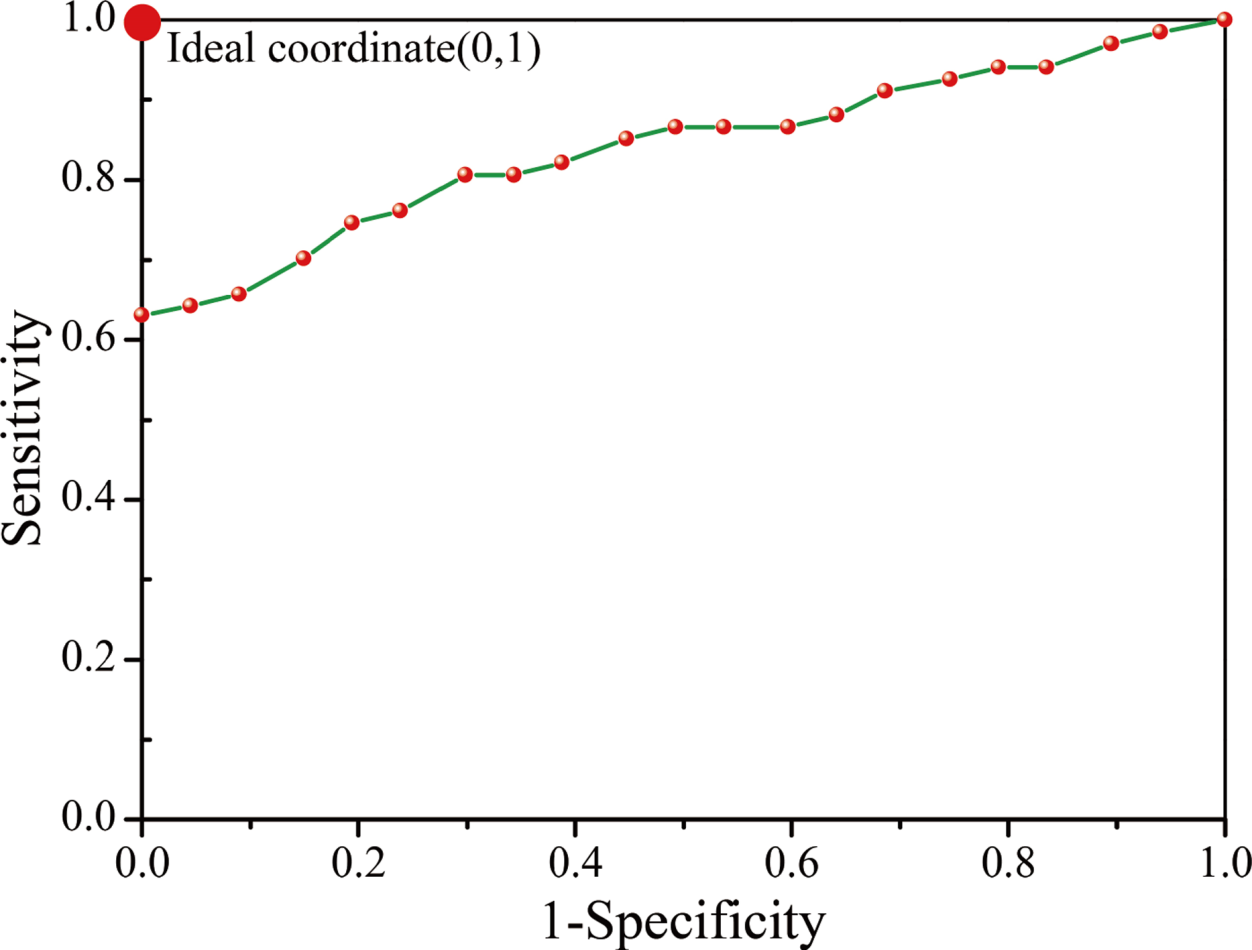
ROC curve for the proposed method for the FUO diagnosis. The red circle in the left-top corner represents the ideal result.

The performance of the proposed method was also quantified on the basis of receiver operating characteristic (ROC) curves. The performance of the differentiating method improved as the curve approached the top left corner. The area under the curve (AUC) is used to quantify the diagnostic ability of a method. The AUC value was calculated to be 0.84 (Fig 9). As shown in Table 6, the proposed diagnostic method for FUO can yield satisfactory results.

**Table 6:** Accuracy classification for different ranges of AUC for the diagnostic test.

AUC Range	Classification
0.9 < AUC < 1.0	Excellent
0.8 < AUC < 0.9	Good
0.7 < AUC < 0.8	Worthless
0.6 < AUC < 0.7	Not good

## Discussion

The basal metabolic activities in the bone differ among individuals, further complicating the issue. Thus, interpretation criteria considerably vary. This study proposed a novel automatic PET/CT image analysis method for FUO diagnosis. We developed the AC-CV model for whole-body bone segmentation from CT images. Then, the corresponding bone structure of CT image was utilized to extract metabolic information from the PET image. The proposed segmentation method is fully automatic and can obtain accurate segmentation of the bone structures. The obtained intensity distribution of the PET and CT images showed that the SUV and PIBGM values in the whole-body bone structures were developed for the computer-assisted diagnosis of FUO. Compared with traditional methods, the proposed method can be used to automatically analyze SUV and PIBGM distribution in the whole bone structures of the patient. The proposed method exhibited a high diagnostic efficiency and an average processing time of 8.75 min per patient.

In this study, we first calculated the mean SUV for the whole bone structures. The mean SUVs of the bone were higher in the FUO patients than in the normal people. However, finding a clear correspondence between SUV distribution and classification is difficult among FUO patients. This result can be attributed to the fact that ^18^F-FDG PET has a low uptake in normal patients but a high uptake in FUO patients because of original or secondary reasons. In infections, the activation of phagocytes, also known as respiratory burst activation, increases ^18^F-FDG PET uptake [27,28]. In non-infectious inflammatory disease, administered ^18^F-FDG PET is mainly taken up by neutrophils and macrophages [29]. A high degree of ^18^F-FDG PET uptake can be observed in neutrophils during the acute phase of inflammation, whereas macrophages and polymorphonuclear leukocytes take up FDG during the chronic phase. A common cause of neoplasm that could lead to FUO is lymphoma [30]. Both BM infiltration and fever may increase the uptake of bone [31]. These phenomena can account for the higher bone FDG metabolism of the FUO patients than that of the normal people.

Basing on the analysis, we divided the extracted bone tissue into two parts: marrow and cortex. The SUV and PIBGM of the cortex and marrow in the whole body were separately calculated. Statistical analysis revealed that the SUV of the bone marrow was about twice that of the cortex and that the SUV of the cortex had a smaller dispersion range than that of the marrow. This result can be attributed to the hemopoietic function of the bone marrow, especially on sites that demonstrate hematopoietic activity, which led to the uptake of FDG in both the normal people and FUO patients. These sites include the sternum, ribs, vertebrae, pelvic bones, proximal femora, and humeri.

Moreover, the SUV and PIBGM of the bone marrow presented slight difference between the FUO patients and normal people as well as among the three etiologies of FUO. The SUV of the cortex can only achieve low-accuracy diagnostic ratio after defining different DANPs. Meanwhile, the PIBGM statistics in the cortex significantly differed between the normal people and FUO patients. The FDG uptake of the cortex can be possibly through the periosteum, a highly vascularized osteogenic organ with a complex and composite structure. The corpora mucosum of the periosteum is the cell source of bone neogenesis and reconstruction. Moreover, it is rich in osteoclasts, mesenchymal stromal stem cells, and vascular endothelial cells. Many studies have shown that vascular endothelial growth factor (VEGF)-stimulated endothelial cells contribute to the total ^18^F-FDG uptake. This phenomenon may be the anatomical basis of FDG uptake in the cortex. The presence of inflammatory cells in FUO patients potentially increases ^18^F-FDG accumulation. The overexpression of VEGF and other stress-related proteins or signaling molecules, such as interleukin-1, transforming growth factor-α, or transforming growth factor-β, may also increase periosteal reaction and tumor infiltration. This phenomenon may lead to the hypermetabolism of the bone cortex among FUO patients. We demonstrated that the proposed method can be used to determine the degree of hypermetabolism and thus classify the etiology of FUO. The proposed method can yield satisfactory results for FUO diagnosis. The mechanisms behind the occurrence of FUO and the increased uptake of FDG in the bone are highly complicated. Identifying the etiology through bone glucose metabolism alone is insufficient. Further investigations should be conducted to identify the cause of bone hypermetabolism. However, the proposed method does not require a professional doctor’s intervention and it can realize automatic analysis of large amounts of PET/CT image data.

## Conclusion

This study proposed an automatic image analysis method for bone glucose metabolism to understand the etiology of FUO. We introduced the concept of PIBGM from bone scan. The SUV of whole bone structures and the PIBGM of the bone cortex significantly differed between the normal and FUO patients. The experimental results demonstrate that the study can achieve automatic classification of FUO patients by the proposed novel biomarker of PIBGM, which has the potential to be utilized in clinical practice. Furthermore, FDG-PET/CT could be a useful standard for the earlier identification of potential patients with FUO, even if the patients have no signs of fever.
